# Phytochemical Characterization and Biological Assessment of *Geranium robertianum* L. Ethanolic Extract on Human Salivary Gland Carcinoma Cells

**DOI:** 10.3390/antiox15030296

**Published:** 2026-02-27

**Authors:** Adina Feher, Adina Căta, Diana Haj Ali, Larisa Bora, Ioana Zinuca Magyari-Pavel, Ana-Maria Vlase, Ștefana Avram, Laurian Vlase, Diana Ungureanu (Similie), Ștefania Dinu, Daliana Minda, Cristina Adriana Dehelean, Mukerrem Betul Yerer, Corina Danciu, Ramona Amina Popovici

**Affiliations:** 1 Doctoral School, “Victor Babeș” University of Medicine and Pharmacy Timișoara, Eftimie Murgu Square, No. 2, 300041 Timișoara, Romania; adina.feher@umft.ro (A.F.); diana.haj-ali@umft.ro (D.H.A.); diana.similie@umft.ro (D.U.); 2 National Institute of Research and Development for Electrochemistry and Condensed Matter, 144 Dr. A. P. Podeanu, 300569 Timişoara, Romania; adina.cata@yahoo.com; 3 Department of Toxicology, Drug Industry, Management and Legislation, Faculty of Pharmacy, “Victor Babeș” University of Medicine and Pharmacy Timișoara, Eftimie Murgu Square, No. 2, 300041 Timișoara, Romania; cadehelean@umft.ro; 4 Research Centre for Pharmaco-Toxicological Evaluation, “Victor Babeș” University of Medicine and Pharmacy Timișoara, Eftimie Murgu Square, No. 2, 300041 Timișoara, Romania; 5 Department of Pharmacognosy-Phytotherapy, Faculty of Pharmacy, “Victor Babeș” University of Medicine and Pharmacy Timișoara, Eftimie Murgu Square, No. 2, 300041 Timișoara, Romania; larisa.bora@umft.ro (L.B.); ioanaz.pavel@umft.ro (I.Z.M.-P.); stefana.avram@umft.ro (Ș.A.); daliana.minda@umft.ro (D.M.); 6 Research and Processing Center of Medicinal and Aromatic Plants, “Victor Babeș” University of Medicine and Pharmacy Timișoara, Eftimie Murgu Square, No. 2, 300041 Timișoara, Romania; 7 Department of Pharmaceutical Botany, Faculty of Pharmacy, Iuliu Hațieganu University of Medicine and Pharmacy, 8 Victor Babeș Street, 400347 Cluj-Napoca, Romania; gheldiu.ana@umfcluj.ro; 8 Department of Pharmaceutical Technology and Biopharmacy, Faculty of Pharmacy, Iuliu Hațieganu University of Medicine and Pharmacy, 8 Victor Babeș Street, 400347 Cluj-Napoca, Romania; laurian.vlase@umfcluj.ro; 9 Department of Pedodontics, Faculty of Dental Medicine, “Victor Babeș” University of Medicine and Pharmacy Timișoara, No. 9, Revolutiei Bv., 300041 Timișoara, Romania; 10 Pediatric Dentistry Research Center, Faculty of Dental Medicine, “Victor Babeș” University of Medicine and Pharmacy Timișoara, No. 9, Revolutiei Bv., 300041 Timișoara, Romania; 11 Department of Pharmacology, Faculty of Pharmacy, Erciyes University, 38039 Kayseri, Turkey; mbyerer@erciyes.edu.tr; 12 Department of Management and Communication in Dental Medicine, Faculty of Dental Medicine, “Victor Babeș” University of Medicine and Pharmacy Timișoara, Eftimie Murgu Square, No. 2, 300041 Timișoara, Romania; ramona.popovici@umft.ro

**Keywords:** *Geranium robertianum* ethanolic extract, phytochemicals, antioxidant, metal analysis, antimicrobial, A253 human salivary gland carcinoma cell line, proliferation, apoptosis

## Abstract

*Geranium robertianum* L. is used in traditional medicine to treat different systemic disorders and holds great therapeutic potential but remains understudied. To this aim, an ethanolic extract obtained from the aerial parts of *G. robertianum* L. (GR) was investigated in terms of phytochemical composition and biological activity. GR extract exhibited high levels of phenolic compounds and flavonoids. The antioxidant activity was determined by means of three different colorimetric assays (DPPH, ABTS, and FRAP), and the results obtained indicate that the ABTS assay showed the highest antioxidant capacity. Metal analysis was also performed. Fe was found to be the most abundant element in the analyzed extract, with a concentration of 363.65 ± 4.18 μg/g, followed by Zn, Mn, Ni, and Cr. Four potentially hazardous heavy metals, As, Co, Pb, and Cd, were found to be under the detection limit. The GR extract exhibited moderate antibacterial activity against both Gram-positive and Gram-negative bacteria, with inhibition zones generally comparable to those of levofloxacin. However, the extract was significantly less effective against the *P. aeruginosa* strain. On A253 human salivary gland carcinoma cells, GR extract elicited a dose-dependent antiproliferative effect, produced morphological changes, and increased ROS and both caspase-3/7 and caspase-9 levels.

## 1. Introduction

The 21st century modern medicine has brought many advancements, and with the help of technology, many diseases have been eradicated or a way to keep them under control. Despite these developments, people increasingly seek solutions to health problems in natural remedies, choosing alternative medicine such as phytotherapy. A wide range of patients choose phytopharmaceutical products over synthetic drugs because, generally, they have better tolerability and a lower risk for side effects [1]. Based on the technology available at this moment, scientists can isolate the natural compounds from a phytocomplex, find their chemical structure, and replicate them synthetically within a laboratory. In some cases it is impossible to replicate the chemical structure because of the complexity of the extracts, making natural sources the only means of obtaining such compounds [2].

One of the medicinal plants that is understudied but holds great therapeutic potential is *Geranium robertianum* L., also known as Herb Robert or Red Robin. It belongs to the *Geraniaceae* family and grows spontaneously in moist and shaded habitats like wastelands, woods, or old walls. *G. robertianum* L. can be found in central and Mediterranean Europe, in temperate parts of Asia, North Africa, North America, and South Africa [3]. Botanically, *G. robertianum* L. is an annual or biennial herbaceous plant, with a height varying between 10 and 60 cm and has green or reddish, long and thin stems. The leaves are light green and triangular in shape, divided into three lobes. It bears two to four violet or pink flowers with a corolla formed of five rounded petals arranged around the ovary [4].

The *Geranium* genus is well-known in terms of its phytochemistry, which is largely characterized by phenolic compounds. Among these, tannins, flavonoids, and phenolic acids are the most extensively researched classes [5]. Phytochemical investigations of *G. robertianum* L. extracts indicate a predominance of hydrolyzable tannins, along with a diverse profile of phenolic acids (ferulic, coumaric, chlorogenic, caffeic, gallic, and ellagic acids), and flavonoids (notably quercetin and kaempferol, present either as glycosides or in aglycone form). In smaller amounts, the extracts also contain lectins, alkaloids, saponins, and organic acids, including malic and citric acids [3,6,7]. Moreover, the aerial part of *G. robertianum* L. contains significant quantities of volatile compounds [8]. The most frequently isolated tannins in *Geranium robertianum* L. are ellagitannins, with the main one being undoubtedly geraniin [9]. The concentration of each active principle depends on the type of extract, the solvent used, and the geographical region where the plant was collected [3,10].

Traditionally, *G. robertianum* L. is used in infusions and decoctions to treat digestive system disorders such as gastritis and diarrhea, or other disorders like tonsillitis, inflammation of the gallbladder, bladder and kidneys, headaches, and nosebleeds. Ethnobotanical research also highlights the varied therapeutic applications of *G. robertianum* L., with different plant parts used to address conditions such as male infertility, rheumatism, liver ailments, high cholesterol, hypertension, and wound healing [11]. Nowadays, the therapeutic properties of *G. robertianum* L. are recognized to be antioxidant, antimicrobial, anti-inflammatory, antidiabetic, neuroprotective, anticancer, and diuretic [3,6].

Since ellagitannins are among the primary compounds found in *G. robertianum* L., highlighting their key pharmacological effects is important. These constituents are recognized for their capacity to exhibit anti-inflammatory and antioxidant effects; they inhibit nitrite and reactive oxygen species production modulating the inflammatory response and protecting the cells from oxidative stress [12]. Geraniin has powerful antioxidant properties but it also has other properties that are less explored such as anti-inflammatory, antihypertensive, apoptotic, antiviral, antidiabetic, antidiarrheal, hepatoprotective, and chemopreventive effects [9].

Regarding the antimicrobial effect of *G. robertianum* L., several types of extracts (methanolic, aqueous, ethanolic, hexane) showed remarkable activities on both Gram-positive and Gram-negative bacteria (*Staphylococcus aureus*, *Streptococcus* sp., *Bacillus cereus*, *Escherichia coli*, *Salmonella infantis*) and fungal (*Candida* sp.) strains [6,13,14].

The antioxidant potential of extracts obtained from *G. robertianum* L. is related to the chemical structure of its constituents, particularly the presence of hydroxyl groups [15]. Evidence from the literature highlights that the solvent selected for extraction significantly influences both the phytochemical profile and the resulting antioxidant performance of the extract. Specifically, hydroalcoholic extracts are known to be highly effective in extracting flavonoid-rich compounds [16]; the antioxidant potential of flavonoids is higher than the antioxidant potential of vitamins C and E [17]. Thus, extracts of *G. robertianum* L. possess marked antioxidant activity [18,19].

Furthermore, several studies have highlighted the cytotoxic potential of this plant in tumor cell lines. Significant results were obtained against cervical, breast, laryngeal, pharyngeal, liver, colon, and non-small cell lung cancer cell lines [6,10,20]. A direct link between the cytotoxic effects and the polyphenol content of the extracts can be taken into consideration. Kaempferol and quercetin have shown anti-cancer properties by increasing autophagy and apoptosis, and decreasing the proliferation, migration, and viability of cancer cells [21]. Additionally, gallic acid, ellagic acid, and geraniin exhibit anticancer effects by inhibiting cell proliferation and triggering apoptosis in tumor cells [22].

*G. robertianum* L. extracts have shown inhibitory activity against a series of enzymes involved in various diseases. Paun et al. demonstrated that the aqueous extract inhibited the urease enzyme. Urease is involved in the virulence of *Helicobacter pylori*, thus in the pathogenesis of ulcers and gastric cancers [23]. Arslan et al. highlighted the acetylcholinesterase inhibitory effect of the aqueous extract, with a possible beneficial role in neurodegenerative conditions such as Alzheimer’s and Parkinson’s diseases [24].

The present study was designed to provide an integrated phytochemical, toxicological, and biological evaluation of a 70% ethanolic extract of *Geranium robertianum* L., obtained by ultrasound-assisted extraction. Specifically, we aimed to: (i) characterize in detail its phytochemical composition using multiple validated LC-MS methods, (ii) assess its antioxidant potential using complementary in vitro assays, (iii) determine its inorganic metal profile to evaluate both nutritional and safety aspects and (iv) investigate its antiproliferative and pro-apoptotic effects on A253 human salivary gland carcinoma cells, including effects on cell viability, clonogenic capacity, ROS production, cytoskeletal organization and caspase-dependent apoptosis.

## 2. Materials and Methods

### 2.1. Extract Preparation and Reagents

The plant material used in this study consisted of the aerial parts of *G. robertianum* L. (GR) (commonly known as Herb Robert), obtained from a licensed herbal pharmacy (Timisoara, Romania). The dried plant material was authenticated by the Department of Pharmacognosy-Phytotherapy. A voucher specimen, 5/GR/PMTM/2025, was deposited in the Herbarium of the Department of Pharmacognosy-Phytotherapy, Faculty of Pharmacy, “Victor Babes” University of Medicine and Pharmacy, Timisoara.

Prior to extraction, the plant material was finely ground into a homogeneous powder. For the preparation of the extract, 10 g of dried plant material were transferred into an Erlenmeyer flask and mixed with 100 mL of 70% ethanol (*v*/*v*) as the extraction solvent. The mixture was subjected to ultrasound-assisted extraction (UAE) using a FALC LBS2 10 LT device (FALC Instruments, Treviglio, Italy) for 60 min in an ultrasonic bath operating at a frequency of 35 kHz and a temperature of 50 °C. Upon completion of the extraction process, the solution was filtered through filter paper using a vacuum pump to remove any solid residues. The resulting filtrate was subsequently concentrated using a rotary evaporator (Laborota 4000 efficient, WB eco, Heidolph Instruments GmbH, Schwabach, Germany) at 50 °C under reduced pressure, until the majority of the solvent was evaporated and a concentrated extract was obtained. The final concentrated extract was stored in airtight containers at a low temperature (−20 °C) until further analysis.

Folin–Ciocâlteu reagent, gallic acid, quercetin dihydrate, sodium nitrite, 1,1-diphenyl-dipicrylhydrazyl (DPPH), 2,4,6-tris(2′-pyridyl)-1,3,5-triazine (TPTZ), Iron (III) chloride (>97%), 2,2′-azino-bis(3-ethylbenzthiazoline-6-sulphonic acid) (ABTS), and sodium acetate trihydrate were obtained from Sigma-Aldrich (St. Louis, MO, USA). Trypsin solution from porcine pancreas (2.5 g/L) and casein Hammcarsten bovine were purchased from Sigma-Aldrich (St. Louis, MO, USA). Sodium hydroxide, aluminum chloride hexahydrate, and trichloroacetic acid (99.5%) were purchased from Merck (Darmstadt, Germany). Trolox 97% was purchased from Acros Organics (Bridgewater, NJ, USA). Ethanol (≥99.8%) and potassium persulfate (≥99%) were acquired from Honeywell (Offenbach, Germany).

### 2.2. Sample Preparation

The dried extract (~0.1 g) was solubilized in 10 mL 70% ethanolic solution. Prior to the analysis, the sample was centrifuged for 20 min at 6000 rpm using a mini centrifuge Labnet C1301 (Labnet International, Edison, NJ, USA) and then was diluted appropriately with 70% ethanol to achieve concentrations within the calibration range. The final results regarding total phenolic content, total flavonoid content, and antioxidant activities were obtained taking into account the dilution factor.

### 2.3. Total Polyphenolic Content

The total phenolic content (TPC) of GR extract was determined according to the Folin–Ciocâlteu method [25], and gallic acid was used as the standard. After incubation for 2 h in the dark, at room temperature, the absorbance of the solutions was measured at 765 nm using a Jasco V 530 UV-Vis spectrophotometer (ABL&E-JASCO, Wien, Austria). The calibration curve (y = 0.001294x + 0.037471, R^2^ = 0.9999) was obtained using gallic acid solutions prepared in the range 25–250 mg/L. All determinations were performed in triplicate, and the results were expressed as mg gallic acid equivalents per g of dried extract (mg GAE/g).

### 2.4. Total Flavonoid Content

The total flavonoid content in GR extract was determined by aluminum chloride colorimetric assay according to a methodology developed starting from the procedures reported previously [26,27]. Briefly, 0.5 mL of the sample or the standard solution was added to a 5 mL volumetric flask containing 2 mL of double-distilled water. To the flask was added 0.15 mL of NaNO_2_ (0.75 M), followed by the addition of 0.15 mL of AlCl_3_·6H_2_O (0.75 M) after 3 min of equilibration. After 3 min, 1 mL of NaOH (1 M) was added, and the final volume was adjusted to 5 mL with double-distilled water. The solutions were mixed and then incubated at room temperature for 30 min in the dark. The absorbance was measured at 351 nm versus a blank prepared with dilution solvent using a Jasco V 530 UV-Vis spectrophotometer (ABL&E-JASCO, Wien, Austria). Quercetin standard solutions (1 to 100 mg/L) in ethanol were used for the calibration curve for total flavonoids (y = 0.0045x + 0.0384, R^2^ = 0.9991). The total flavonoid content was expressed in mg quercetin equivalents per g of dried extract (mg QE/g). All determinations were performed in triplicate.

### 2.5. Phytochemical Analysis

The phytochemical profile of the extract was determined using five distinct, previously validated LC-MS/MS analytical methods [28,29,30,31]. The analyses were performed on an Agilent 1100 HPLC system (Agilent Technologies, Santa Clara, CA, USA) equipped with a UV detector (Agilent Technologies, Santa Clara, CA, USA) and coupled to an Agilent Ion Trap 1100 SL mass spectrometer (Agilent Ion Trap 1100 SL mass spectrometer). Data acquisition and processing were performed using Agilent ChemStation (vB01.03) and DataAnalysis (v5.3) software.

The first analytical method was employed for the simultaneous identification and quantification of 23 phytochemicals (phenolic acids and flavonoids). Chromatographic separation was achieved on a Zorbax SB-C18 column (100 mm × 3.0 mm i.d., 3.5 µm; Agilent Technologies, Santa Clara, CA, USA). Chromatographic separation was achieved under gradient conditions using methanol and 0.1% aqueous acetic acid (*v*/*v*) as mobile phases, delivered at a flow rate of 1 mL/min. Detection was carried out with a mass spectrometer equipped with an electrospray ionization source operating in negative ion mode. Full experimental details were previously described by Vlase A-M et al. [28], Solcan M-B et al. [29,30], and Safta DA et al. [31].

A second validated LC-MS/MS method was employed to identify eight additional phenolic acids and catechins (gallic acid, protocatechuic acid, vanillic acid, syringic acid, catechin, epicatechin, epigallocatechin, and epigallocatechin gallate). The analysis was carried out on the same chromatographic system and column, while applying an adjusted gradient elution program and comparable ionization settings [28,29,30,31]. In this approach, methanol and 0.1% aqueous acetic acid (*v*/*v*) were again used as mobile phases, with the separation profile adapted through modification of the gradient scheme. Detection was performed using the ESI source in negative mode, as previously detailed [28,29,30,31].

The analysis of procyanidins (B1, B2, C1, and A2) was performed by a dedicated LC-MS/MS method employing the same equipment and column. Separation was performed on the Zorbax SB-C18 column (100 mm × 3.0 mm i.d., 3.5 µm; Agilent Technologies, Santa Clara, CA, USA) with a gradient of methanol and 0.1% aqueous acetic acid (*v*/*v*) at 1 mL/min. Analyte detection was conducted in negative ionization mode using ESI-MS/MS. The detailed methodology was previously described by Solcan M-B et al. [29,30] and Safta DA et al. [31].

Determination of phytosterols (ergosterol, stigmasterol, campesterol, β-sitosterol) was performed by using a validated LC-MS/MS analytical method with isocratic elution (acetonitrile–methanol, 90:10, *v*/*v*, at 1 mL/min) on the same analytical column. The mass spectrometer was equipped with an atmospheric pressure chemical ionization (APCI) source operating in positive mode. Quantification was performed in multiple-reaction monitoring (MRM) mode, as previously described [28,31].

Finally, tocopherols (α-, γ-, δ-forms) were quantified using an LC-MS/MS method operating under isocratic conditions (Zorbax SB-C18, 100 mm × 3.0 mm i.d., 3.5 µm; Agilent Technologies, Santa Clara, CA, USA) with water–methanol (7:93, *v*/*v*) as the mobile phase (1 mL/min). Detection was achieved using an APCI source in negative ionization, operating in MRM mode [28,31].

### 2.6. Antioxidant Activity Evaluation

#### 2.6.1. DPPH Assay

The DPPH assay was carried out according to the procedure described by Brand-Williams et al. [32] with some modifications. Briefly, 0.1 mL of the sample was added to 2.9 mL of freshly prepared 0.09 mM DPPH in methanol. The solution was mixed and then incubated for 2 h in the dark at room temperature. The decrease in absorbance after 2 h was measured at 515 nm using a Jasco V 530 UV-Vis spectrophotometer (ABL&E-JASCO, Wien, Austria). Trolox was used as an antioxidant reference compound. The calibration curve (y = 70.7158x + 5.8592, R^2^ = 0.9935) was obtained using standard solutions in the range 0.1–0.6 mmol/L Trolox. The antioxidant activity was expressed as mmol Trolox equivalent per g of dried extract (mmol TE/g). The sample was analyzed in triplicate.

#### 2.6.2. ABTS Assay

ABTS radical cation was generated through the reaction between ABTS (7 mmol/L) and potassium persulfate (2.45 mmol/L) in sodium acetate buffer, pH 4.5 [33]. The dark blue-green radical solution was incubated in the dark at room temperature for 16–18 h, and then the solution was diluted to adjust the absorbance value to ~1.0 at 734 nm. 0.1 mL of the sample and 2.9 mL of ABTS solution were mixed and then incubated for 2 h in the dark at room temperature. The absorbance decrease was measured at 734 nm using a Jasco V 530 UV-Vis spectrophotometer (ABL&E-JASCO, Wien, Austria). A calibration curve (y = 67.8729x + 3.3698, R^2^ = 0.9991) was obtained using Trolox as a reference compound in the concentration range of 0.1–0.6 mmol/L. The antioxidant activity was expressed as mmol Trolox equivalent per g of dried extract (mmol TE/g). The sample was analyzed in triplicate.

#### 2.6.3. FRAP Assay

The FRAP (ferric reducing/antioxidant power) assay was performed according to Benzie and Strain [34], with some modifications. The FRAP reagent was prepared by mixing sodium acetate buffer (300 mM, pH 3.6), TPTZ solution (10 mM in 40 mM HCl), and FeCl_3_ solution (20 mM) in a 10:1:1 (*v*/*v*/*v*) ratio. The resulting solution was diluted with 2 volumes of double-distilled water and then incubated at 37 °C for 30 min. The FRAP reagent (2.9 mL) and sample solution (0.1 mL) were thoroughly mixed and then incubated in the dark for 2 h at room temperature. The absorbance was measured at 593 nm using a Jasco V 530 UV-Vis spectrophotometer (ABL&E-JASCO, Wien, Austria). The calibration curve (y = 1.3409x + 0.0171, R^2^ = 0.9973) was prepared using different concentrations of Trolox in the range 0.1–0.6 mmol/L. The antioxidant activity was expressed as mmol Trolox equivalent per g of dried extract (mmol TE/g). The sample was analyzed in triplicate.

### 2.7. Sample Preparation and Metal Analysis by Atomic Absorption Spectroscopy

Approximately 50 mg of GR extract was digested with 5.0 mL of 67% HNO_3_ (Sigma Aldrich, St. Louis, MO, USA) in DAP-60K high-pressure Teflon vessels using a microwave digestion system (MWS-2, Berghof, Eningen, Germany) operated under a three-stage program (Table 1). Following digestion, the mixture was filtered to remove residual solids, and the filtrate was quantitatively adjusted to 20 mL with ultrapure water (EASYpureRoDi^®^ apparatus, Barnstead, Dubuque, IA, USA).

Elemental concentrations were quantified by graphite furnace atomic absorption spectrometry using a novAA 400G instrument (Analytik Jena, Jena, Germany) equipped with an MPE60 autosampler, applying the manufacturer’s recommended analytical program (Cookbook) specific for each element. Data acquisition and processing were carried out with WinAAS 3.17.0 software. When analyte levels exceeded the calibration range, appropriate dilutions were made using 0.5% HNO_3_ prior to measurement. All samples were analyzed in six replicates.

Calibration curves for each element were established in advance at their corresponding analytical wavelengths (Table 2). Standards were prepared by serial dilution of 1000 mg/L CertiPUR^®^ stock solution (Merck, Darmstadt, Germany) with ultrapure water.

### 2.8. Antimicrobial Activity Evaluation

Antimicrobial activity determination was done as recommended by the Clinical Laboratory and Standards Institute (CLSI), and the other studies [35,36,37].

#### 2.8.1. Bacterial Strains

The antimicrobial potential of GR extract was evaluated against five reference bacterial strains (Thermo Scientific, Waltham, MA, USA): *Streptococcus mutans* ATCC 35668, *Streptococcus pyogenes* ATCC 19615, *Staphylococcus aureus* ATCC 25923, *Escherichia coli* ATCC 25922, and *Pseudomonas aeruginosa* ATCC 27853.

#### 2.8.2. Disk Diffusion Method

The microbial suspensions were adjusted with physiological saline to a concentration of 0.5 McFarland and 100 µL of these suspensions was placed on the surface of Mueller-Hinton agar (MH) or MH supplemented with sheep blood (Thermo Scientific, Loughborough, UK). 10 µL from GR were added to a blank paper disk (BioMaxima, Lublin, Poland), then deposited on the surface of the MH plates inoculated with the microbial suspensions and were incubated at 35 °C for 24 h. The reading of the inhibition zones was made in mm. All tests were done in triplicate for each tested strain. Levofloxacin (Biomaxima, Lublin, Poland) was used as a positive control, and as a negative control, an unimpregnated disk was used.

#### 2.8.3. The Broth Dilution Method—Determination of Minimum Inhibitory Concentration (MIC)

In four test tubes, serial two-fold dilutions of the tested sample (25, 12.5, 6.25, 3.125 mg/mL) in Mueller-Hinton broth (Thermo Scientific, Loughborough, UK) were made and added with the microbial inoculum (5 × 10^5^ bacteria/mL). After incubating the test tubes at 35 °C for 24 h, the MIC (the lowest concentration without visible growth) was determined.

### 2.9. Cellular Viability Evaluation

The cell viability assessment was performed using the MTT technique, described by Apostolova et al. [38]. Five concentrations of GR extract (20, 50, 100, 150, and 200 µg/mL) were evaluated on A253 (human submaxillary salivary gland carcinoma) cells (HTB-41™, American Type Culture Collection, ATCC, Rockville, MD, USA). The cells were cultured in 96-well plates (1 × 10^4^/well), and after 72 h of treatment, a volume of 100 µL of fresh medium and a volume of 10 µL/well MTT reagent (Sigma-Aldrich, St. Louis, MO, USA) were added and incubated for 3 h at 37 °C and 5% CO_2_. Then, 100 µL/well of solubilization solution was added, and further incubated at room temperature for 30 min in a dark place. The absorbance was determined at 570 and 630 nm by means of Cytation 5 (BioTek Instruments Inc., Winooski, VT, USA).

### 2.10. Cellular Morphology Assessment

Morphological alterations in A253 cells were examined following their cultivation in 96-well plates at a seeding density of 1 × 10^4^ cells per well. Thus, morphological alterations after 72 h of GR extract exposure (at 20, 50, 100, 150, and 200 µg/mL) were assessed using brightfield microscopy at 20× magnification with the Lionheart FX automated imaging system (BioTek Instruments Inc., Winooski, VT, USA). Subsequently, the images were captured and analyzed using Gen5™ Microplate Data Collection and Analysis Software (version 3.14; BioTek Instruments Inc., Winooski, VT, USA) [39].

### 2.11. Colony Formation Assay

To investigate the capacity of GR extract (20, 50, 100, 150, and 200 µg/mL) to suppress clonogenic growth in A253 cells, the colony formation assay was performed in accordance with Marcovici et al. [40]. Briefly, cells were seeded at a low density of 100 cells per well in 96-well plates and allowed to adhere. Subsequently, they were exposed to the indicated concentrations of GR extract for 72 h, after which the culture medium was periodically replaced with fresh medium for a duration of 7–10 days. At the end of the incubation period, colonies were fixed with 4% paraformaldehyde (Santa Cruz Biotechnology, Dallas, TX, USA) and stained with 0.2% crystal violet (Electron Microscopy Sciences, Hatfield, PA, USA) solution in phosphate-buffered saline (PBS) (Sigma-Aldrich Merck KGaA, Darmstadt, Germany) for 10 min at room temperature. After rinsing with distilled water and capturing representative images, the colonies were solubilized with 1% sodium lauryl sulphate (SLS) (Sigma-Aldrich Merck KGaA, Darmstadt, Germany), and absorbance was measured at 550 nm using a Cytation 5 imaging reader (BioTek Instruments Inc., Winooski, VT, USA). The number of colonies formed was determined by microscopic evaluation.

### 2.12. ROS Production

In order to evaluate the impact of the GR extract at 20, 50, 100, 150, and 200 µg/mL on intracellular ROS production in A253 cells, the experiment was carried out in accordance with the protocol described by Talpoș et al. [39] and following the manufacturer’s recommendations [41]. After 66 h of incubation, A253 cells cultured in opaque white 96-well plates were treated with 20 µL of ROS-Glo™ H_2_O_2_ Substrate Solution added to each well. The plates were then returned to the incubator and maintained at 37 °C and 5% CO_2_ until the 72 h treatment period was completed. Next, 100 µL of ROS-Glo™ Detection Solution was added to each well, followed by incubation for 20 min at room temperature, protected from light. The ROS-Glo™ kit was provided by Promega (Madison, WI, USA). Finally, luminescence was recorded using a Cytation 5 instrument (BioTek Instruments Inc., Winooski, VT, USA), and the results were expressed relative to the control group, represented by untreated cells.

### 2.13. Immunofluorescence Imaging of Nuclei and F-Actin Filaments

To determine the impact of the GR extract on cell nuclei and F-actin filaments, the cells were seeded into black-walled, with a clear bottom 96-well plates and exposed to increasing concentrations of the extract (20, 50, 100, 150, and 200 µg/mL) for 72 h. Following treatment, the culture medium containing the extract was discarded, and the cells were gently rinsed with PBS. Then, the cells were stained with 100 µL/well of Hoechst 33342, purchased from Thermo Fisher Scientific (Waltham, MA, USA) (1:2000 dilution in PBS) and incubated for 10 min at room temperature, protected from light. Subsequently, cells were washed three times with PBS. After that, the cells were fixed with 4% paraformaldehyde in PBS for 10 min at room temperature, followed by permeabilization with 2% Triton X-100 (Sigma-Aldrich, Merck KGaA, Darmstadt, Germany) in PBS for 30 min. To block nonspecific binding, cells were incubated with 30% fetal bovine serum (FBS) obtained from ATCC (Manassas, VA, USA) and prepared in 0.01% Triton X-100 solution for 1 h at room temperature. Subsequently, cells were incubated overnight at 4 °C with Rhodamine Phalloidin (Biotium, Fremont, CA, USA) diluted 1:200 in PBS for actin filament visualization. The next day, cells were washed three times with 0.01% Triton X-100 in PBS before microscopic observation. Immunofluorescence images were acquired using a Nikon Eclipse Ji fluorescence microscope (Tokyo, Japan) under standardized settings. A similar protocol was described by Marcovici et al. [40].

### 2.14. Caspase 3/7 and Caspase 9 Activity Evaluation

A253 cells (1 × 10^4^ cells/well) were seeded in white, opaque 96-well plates and incubated with GR extract at final concentrations of 20, 50, 100, 150, and 200 µg/mL for 72 h. Once the treatment interval was completed, the plates were left at room temperature for 30 min to equilibrate. Then, 100 µL of Caspase-Glo^®^ 3/7 or Caspase-Glo^®^ 9 reagent (Promega, Madison, WI, USA), prepared according to the manufacturer’s instructions, was added to each well. Next, the plates were gently shaken on an orbital shaker for 30 s, followed by a 1 h incubation at room temperature, protected from light. Afterwards, the luminescence values were recorded using a Cytation 5 multi-mode reader (BioTek Instruments Inc., Winooski, VT, USA), with untreated cells serving as the control group. The experiment was performed following a procedure similar to that described by Talpoș et al. [39].

### 2.15. Statistical Analysis

Statistical evaluation of the data was performed using GraphPad Prism software (version 10.2.3, GraphPad Software, San Diego, CA, USA, www.graphpad.com, accessed on 11 November 2025). Differences between GR extract-treated groups and the control were assessed by one-way ANOVA followed by Dunnett’s multiple comparison test. Statistical significance was denoted as follows: * *p* < 0.05, ** *p* < 0.01; *** *p* < 0.001; **** *p* < 0.0001.

## 3. Results

### 3.1. Phytochemical Composition

To analyze the phytochemical profile of GR ethanolic extract, total polyphenolic content, total flavonoid content, and LC-MS analysis were employed.

The TPC analysis revealed significant amounts of polyphenolic compounds (Table 3). The TPC analysis showed a value of 191.737 ± 5.828 mg GAE/g dried extract, while the TFC reached 117.487 ± 8.389 mg QE/g dried extract.

GR extract was particularly rich in polyphenolic compounds (especially gallic and protocatechuic acid), while small amounts of syringic and vanillic acids were also registered (Table 4). Procyanidin B3, B4, B2, C2, C1, and A1 were undetected, while procyanidin B1 was present in negligible amounts (0.0069 ± 0.0005 mg/g dried extract), indicating limited oligomeric flavonoid content in GR. δ-tocopherol (0.00170 ± 0.00001 mg/g dried extract) was the only detectable isoform in GR extract, indicating a modest vitamin E content. Further, significant amounts of sterolic components were found in the tested sample, with β-sitosterol as being the most abundant compound, followed by campesterol and stigmasterol. As seen in Table 4, flavonoids such as hyperoside (4.272 ± 0.299 mg/g dried extract), isoquercitrin (2.694 ± 0.107 mg/g dried extract), quercetin (1.224 ± 0.036 mg/g dried extract), and quercitrin (0.291 ± 0.02 mg/g dried extract) were also detected.

Regarding the presence of phenolic acids, the following compounds were predominant: chlorogenic acid (0.77 ± 0.023 mg/g dried extract), ferulic acid (0.681 ± 0.027 mg/g dried extract), and *p*-coumaric acid (0.627 ± 0.031 mg/g dried extract).

Detailed chromatographic data, including UV profiles for polyphenols quantified by the first LC-MS method and TICs for all other analyzed compounds (additional phenolic acids and catechins, tocopherols, and sterols), are available in the Supplementary Material.

### 3.2. Antioxidant Activity

The antioxidant activity of GR extract was evaluated using three distinct assays: DPPH, ABTS, and FRAP. Among these methods, the ABTS assay showed the highest antioxidant capacity, while the DPPH and FRAP assays resulted in lower antioxidant activity values (Table 5).

### 3.3. Determination of Inorganic Elements

The content of ten metals was determined in the dry extract of GR by atomic absorption spectroscopy, and the obtained results are shown in Table 6. The concentration values of detected metals were in the following descending order: iron (Fe) > zinc (Zn) > manganese (Mn) > nickel (Ni) > chromium (Cr). The values for copper (Cu) and four heavy metals with known toxic potential, namely arsenic (As), cobalt (Co), lead (Pb), and cadmium (Cd), were found to be under the detection limit.

### 3.4. Antimicrobial Effect

The GR extract was tested against both Gram-positive (*S. mutans*, *S. pyogenes*, and *S. aureus*) and Gram-negative bacteria (*E. coli* and *P. aeruginosa*) in Table 7. GR displayed inhibition zones similar to those of levofloxacin, which served as a positive control, with a MIC value of 25 μg/mL. However, the sample was significantly less effective against the *P. aeruginosa* strain, with a small inhibition zone of 7 mm and no MIC value determined. In contrast, levofloxacin exhibited a 20 mm inhibition zone, indicating greater efficacy. Overall, GR extract demonstrated a more potent antimicrobial effect against Gram-positive bacterial strains compared to the Gram-negative ones.

### 3.5. Cell Viability Assay

**Figure 1:**
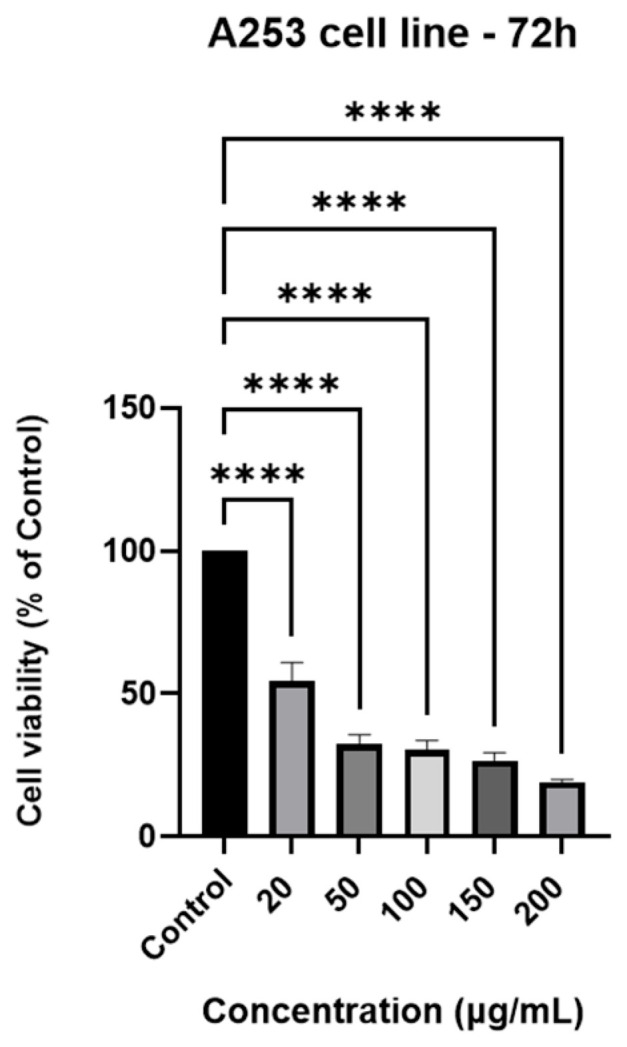
A graphic illustration of A253 human salivary gland carcinoma cells viability after 72 h treatment with GR extract (20, 50, 100, 150, and 200 µg/mL). The results are shown as a percentage (%) normalized to the control. To analyze statistical differences between control and treatment groups, the one-way ANOVA test was followed by Dunnett’s multiple comparison post-test. “*” marks statistical significance (**** *p* < 0.0001).

For the purpose of evaluating the cell viability of the A253 cell line after 72 h of treatment, the MTT assay was performed. The data indicated a decrease in cell viability of A253 human salivary gland carcinoma cells in a dose-dependent manner; the highest decrease in viability (19.20%) was observed at the concentration 200 µg/mL of GR extract (Figure 1).

### 3.6. Cellular Morphology Assessment

**Figure 2:**
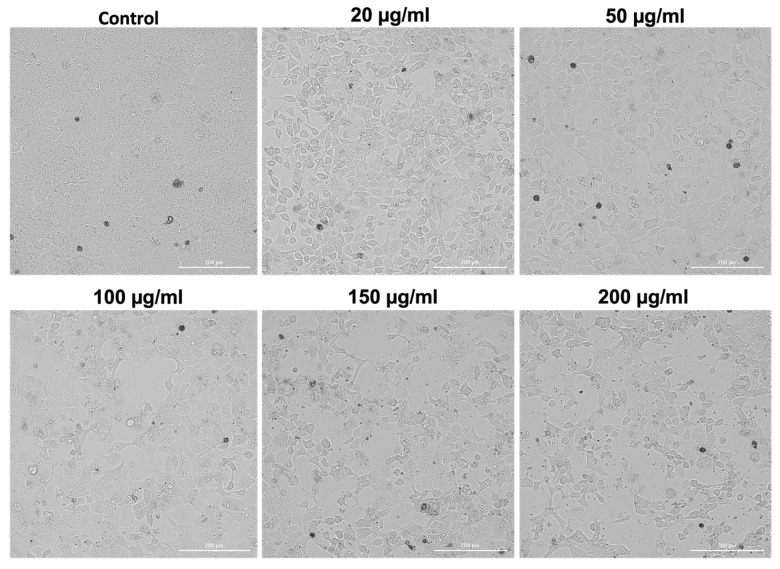
The morphological appearance of A253 human salivary gland carcinoma cells after 72 h treatment with GR extract at different concentrations of 20, 50, 100, 150, and 200 µg/mL. The scale indicates 200 µm.

The morphology of A253 cells was significantly affected in a dose-dependent manner following 72 h treatment with the GR extract (Figure 2). With increasing concentrations, salivary gland carcinoma cells gradually exhibited a more elongated appearance, consistent with cytoplasmic shrinkage. At the highest dose, these alterations were the most evident, accompanied by signs of mild cellular fragmentation.

### 3.7. Colony Formation Assay

**Figure 3:**
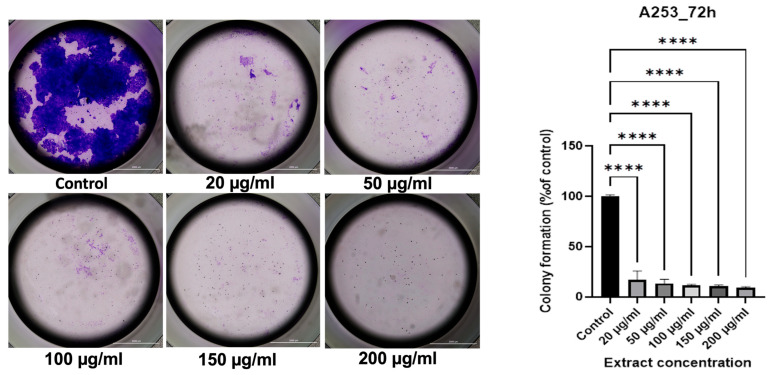
Colony formation assay of A253 human salivary gland carcinoma cells treated with different concentrations of GR extract (20, 50, 100, 150, and 200 µg/mL). Colonies were stained with 0.2% crystal violet. Representative images of colonies (**left**) and quantification expressed as % of control (**right**) are shown. The results are shown as a percentage (%) normalized to the control. The scale indicates 2000 µm. To analyze statistical differences between control and treatment groups, the one-way ANOVA test was followed by Dunnett’s multiple comparison post-test. “*” marks statistical significance (**** *p* < 0.0001).

The ability of A253 cells to form colonies was markedly inhibited by 72 h treatment with GR extract in a concentration-dependent manner (Figure 3). When expressed as a percentage of control, colony formation was reduced to 16.85%, 13.46%, 11.46%, 10.49%, and 9.00% at 20, 50, 100, 150, and 200 µg/mL, respectively. These findings demonstrated a strong suppressive effect of the extract on the clonogenic potential of A253 cells, with the highest concentration producing the most pronounced inhibition.

### 3.8. ROS Production

**Figure 4:**
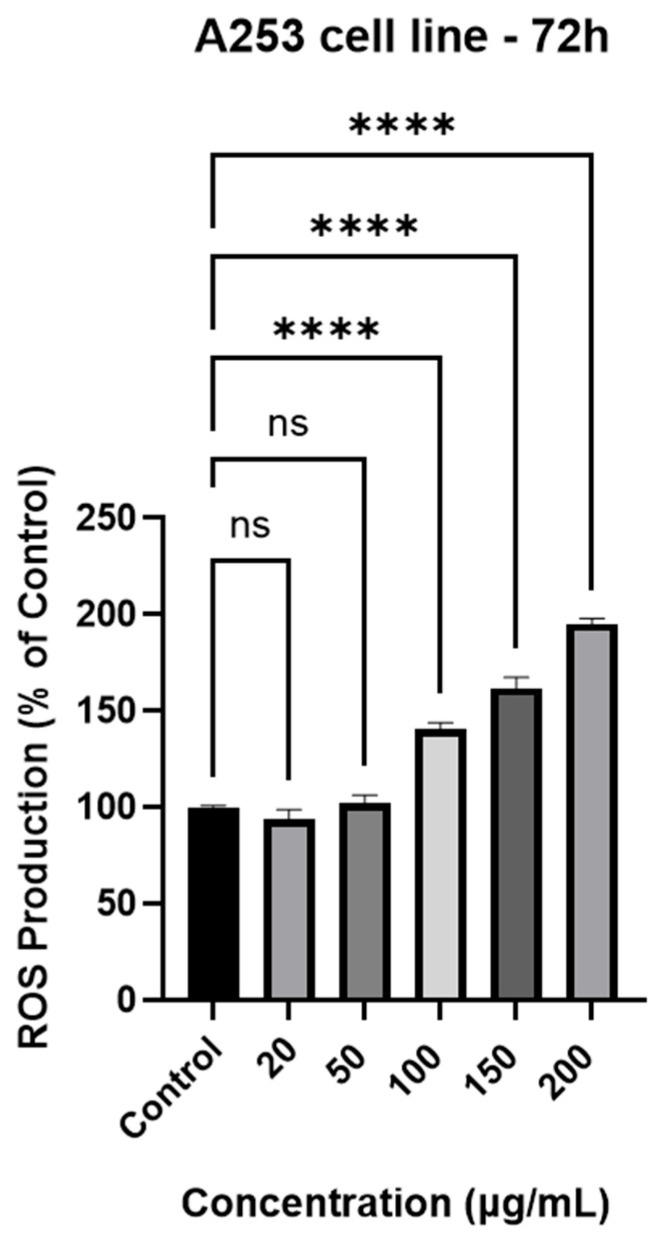
The impact of 72 h exposure to GR extract at different concentrations (20, 50, 100, 150, and 200 µg/mL) on ROS production in A253 human salivary gland carcinoma cells. The results are shown as a percentage (%) normalized to the control. To analyze statistical differences between control and treatment groups, the one-way ANOVA test was followed by Dunnett’s multiple comparison post-test. “*” marks statistical significance (ns: not significant, **** *p* < 0.0001).

Following 72 h of treatment with GR extract, the impact of the lower concentrations (20 and 50 µg/mL) on ROS production was comparable to that of the control group (93.67% and 102.01%, respectively) (Figure 4). A more pronounced increase was observed at 100 µg/mL (140.33%), while at 150 µg/mL and 200 µg/mL the ROS levels reached 161.02% and 195.24% of the control, respectively.

### 3.9. Immunofluorescence Imaging of Nuclei and F-Actin Filaments

**Figure 5:**
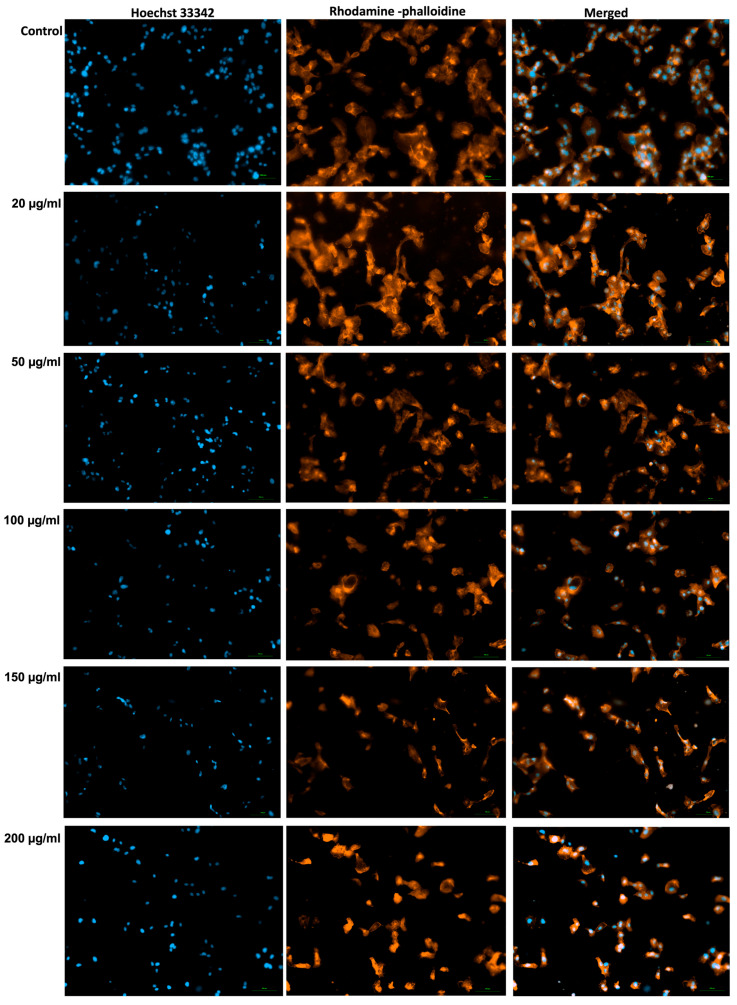
Nuclei and F-actin aspect of A253 human salivary gland carcinoma cells after 72 h of exposure to GR extract at 20, 50, 100, 150, and 200 µg/mL. The scale indicates 100 µm. Nuclei were stained with Hoechst 33342 (blue), F-actin was stained with Rhodamine-phalloidin (red-orange). The merged images show the overlay of both channels.

Immunofluorescence staining was performed using Hoechst 33342 to visualize nuclei and Rhodamine–phalloidin to label filamentous actin (F-actin) (Figure 5). The 72 h treatment of A253 cells with GR extract induced clear dose-dependent morphological changes. Hoechst staining showed progressive nuclear condensation and increased fluorescence intensity, together with a reduction in nuclear size at higher concentrations, suggesting chromatin compaction. In parallel, Rhodamine–phalloidin staining revealed enhanced F-actin signal but with reduced cellular spreading, giving the cells a smaller and more compact morphology. These alterations were most evident at the highest concentration, consistent with nuclear damage and cytoskeletal remodeling typically associated with apoptosis.

### 3.10. Caspase-3/7 and Caspase-9 Activity Evaluation

**Figure 6:**
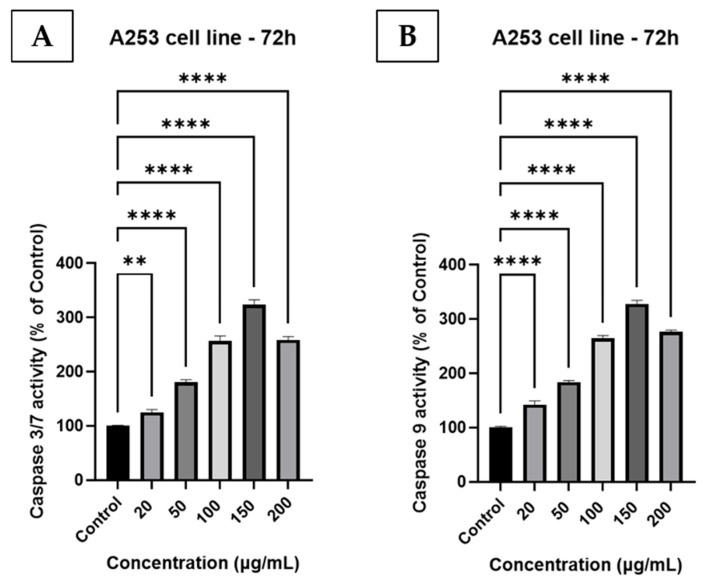
The impact in A253 human salivary gland carcinoma cells of 72 h exposure to GR extract at 20,50, 100, 150, and 200 µg/mL on (**A**). the caspase-3/7 and (**B**). caspase-9 levels. The results are shown as a percentage (%) normalized to the control. To analyze statistical differences between control and treatment groups, the one-way ANOVA test was followed by Dunnett’s multiple comparison post-test. “*” marks statistical significance (** *p* < 0.01; **** *p* < 0.0001).

Exposure of A253 cells to GR extract for 72 h resulted in a dose-dependent activation of both caspase-3/7 and caspase-9 (Figure 6). Compared with untreated controls (set at 100%), caspase-3/7 activity increased to 125.25%, 180.11%, 256.66%, 323.01%, and 258.33% at 20, 50, 100, 150, and 200 µg/mL, respectively. A similar trend was observed for caspase-9, with values of 142.33%, 183.23%, 264.50%, 327.60%, and 276.26% at the same concentrations. The maximal induction for both caspase-3/7 and caspase-9 was observed at 150 µg/mL, followed by a slight decrease at 200 µg/mL, while remaining well above control levels.

## 4. Discussion

*Geranium robertianum* L. has a long history of use in traditional medicine and has demonstrated promising therapeutic effects across various pathologies, while also being explored in studies related to cancer. The limited number of studies available in the scientific literature highlights the need for further investigation of its therapeutic effects and underlying mechanisms. In the present study, we investigated the phytochemical composition and biological effects of an ethanolic extract obtained from *Geranium robertianum* L.

The LC-MS analysis detailed and quantified various polyphenols, procyanidins, tocopherols, sterols, and flavonoids. GR extract exhibited high levels of phenolic compounds (191.737 ± 5.828 mg GAE/g dried extract) and flavonoids (117.487 ± 8.389 mg QE/g dried extract). In addition, the extract was found to contain several lipophilic compounds, such as β-sitosterol (5.391 ± 0.269 mg/g dried extract) and δ-tocopherol (0.00170 ± 0.00001 mg/g dried extract). In this context, previous studies have focused on identifying and quantifying the phytocompounds found in various GR extracts. Mekinić et al. obtained and analyzed an 80% ethanolic extract from Croatian GR aerial parts. The Folin–Ciocâlteu method revealed that this extract possessed 119.6 ± 0.5 mg GAE/g polyphenolic compounds. Moreover, the HPLC analysis showed the presence of only one polyphenolic acid (gallic acid in a concentration of 0.20 ± 0.01 mg/g), but also the presence of more flavonoids (epicatechin 0.45 ± 0.04 mg/g, rutin 2.13 ± 0.10 mg/g, and quercetin-4-glucoside 0.81 ± 0.08 mg/g) [13]. Furthermore, Paun et al. examined and compared the properties of both aqueous and aqueous-alcoholic extracts from Romanian GR leaves. The total phenolic content in the initial and microfiltrate hydroalcoholic extracts exceeded 800 mg GAE/L, while the total phenolic content in the homologous aqueous extracts was approximately 700 mg GAE/L. The hydroalcoholic microfiltrate contained the highest levels of caffeic acid (20.18 mg/kg), while kaempferol was the most representative flavonoid (284.57 mg/kg) [42]. The data revealed that the aqueous-alcoholic extracts contained higher levels of phenolic acids and flavonoids compared to the aqueous extracts, confirming that the organic solvents extracted polyphenolic compounds more efficiently.

According to the experiments performed by Piwowarski and colleagues, an aqueous lyophilized extract from GR aerial parts displayed a total polyphenol content of 26.9 ± 1.2% and a total tannin content of 20.7 ± 1.3% [43]. This extract was studied for its ability to inhibit hyaluronidase and elastase, two enzymes associated with inflammatory skin diseases. Another study conducted by Bawish et al. investigated the phytochemical composition of an aqueous extract of GR leaves from Egypt. The research group observed that this extract contained 194.96 ± 1.24 mg GAE/g polyphenols, which is a value similar to that of the ethanolic extract (191.737 ± 5.828 mg GAE/g dried extract) analyzed within this study. The LC/MS/MS analysis revealed the presence of several polyphenolic acids, including chlorogenic acid (443.84 µg/g), gallic acid (146.87 µg/g), coumaric acid (7.08 µg/g), caffeic acid (3.11 µg/g), ellagic acid (115.65 µg/g), syringic acid (7.58 µg/g), dihydroxybenzoic acid (29.95 µg/g), and ferulic acid (5.46 µg/g) [44]. Although geraniin was not directly quantified due to analytical limitations, the high gallic acid content, strong antioxidant activity, and ROS-mediated apoptosis observed in A253 cells are consistent with previously reported biological effects of geraniin-containing extracts. Comparison between the aqueous extract reported by Bawish et al. [44] and the ethanolic one investigated in this study revealed differences in the distribution of polyphenolic acids. The lyophilized aqueous extract additionally contained caffeic, ellagic, and dihydroxybenzoic acids, whereas the ethanolic extract was the only one that contained protocatechuic and vanillic acids. Furthermore, the flavonoid compositions varied between the two extracts. The aqueous extract had a remarkable quantity of rutin, measuring 230.84 µg/g, while the amount of rutin in the ethanolic extract, evaluated in this study, was below the limit of quantification. Conversely, the ethanolic extract contained apigenin, which was not detected in the aqueous extract [44]. The high content of polyphenols contributes to protection against oxidative stress and inflammation, as these compounds can scavenge free radicals. The variations in the phytochemical composition may be attributed to pedoclimatic conditions. Differences in extraction methodologies may also contribute to these discrepancies [45,46]. The physicochemical characteristics and compositional analysis of an extract can influence the biological response, highlighting the importance of detailed compositional profiling when interpreting cellular response [47,48].

As an overview, Swiatek et al. used commercially available dried herb of GR (Grodzisk, Poland) to prepare four types of extracts, namely methanolic, aqueous, hexane, and ethyl acetate. Phenolic acids, tannins, flavonoids, and fatty components were primarily detected. The phenolic compounds included the quinic forms of coumaric, ferulic, and caffeic acids, with tannins characterized by gallic and ellagic acids, while flavonoids were represented by quercetin and kaempferol-based structures [6].

The antioxidant activity was determined for the analyzed GR ethanolic extract using three different colorimetric assays: DPPH, ABTS, and FRAP. Each assay reflects different antioxidant mechanisms. The DPPH and ABTS assays measure the ability of the tested compounds to transfer electrons or hydrogen atoms to the radicals DPPH and ABTS·^+^, effectively neutralizing them. In contrast, the FRAP assay assesses the reducing power of these compounds by determining their capacity to convert ferric ions (Fe^3+^) into ferrous ions (Fe^2+^) [49]. The ABTS assay revealed the highest antioxidant capacity, with a value of 3.159 mmol TE/g dried extract. The DPPH assay exhibited an antioxidant activity of 2.091 mmol TE/g dried extract, closely followed by the FRAP assay, which presented the lowest value of 2.061 mmol TE/g dried extract. However, the DPPH and FRAP results were quite similar, suggesting comparable reducing capacities in these two assays.

The potential to reduce free radicals has been studied by many other researchers for various types of GR extracts. For example, a dry ethanolic extract from aerial parts of Turkish GR revealed an impressive 90.71 ± 0.77% scavenging effect at a concentration of 2000 μg/mL during the DPPH test [50]. Regarding the aqueous extracts, a concentrated aqueous extract of *G. robertianum* L. obtained by Paun et al. determined a 92.9% DPPH absorption inhibition [51]. Similarly, the aqueous extract of the whole GR plant, obtained by Graça et al., showed an EC_50_ value of 65 ± 1 µg/mL within the DPPH assay, having a total phenolic content of approximately 228 mg GAE/g [10,52]. The 6% GR aqueous extract obtained by Neagu et al. through microfiltration was investigated in terms of its free radical scavenging potential. The DPPH and ABTS tests displayed total equivalent antioxidant capacity (TEAC) values of 0.16902 ± 0.00686 mmol Trolox/g and 0.53956 ± 0.01121 mmol Trolox/g, respectively [20]. These results suggested that the concentrated aqueous extract displayed significantly lower radical scavenging activity than the ethanolic one investigated in the current study. The decoctions made from the leaves and stems of Portuguese GR, as reported by Catarino and colleagues, exhibited high antioxidant potential. The DPPH, ABTS, and FRAP tests demonstrated that the leaf extract showed stronger radical scavenging activity compared to the stem extract, although it was still weaker than the standard controls, which were vitamin C and butylated hydroxytoluene (BHT). The IC_50_ values for the leaf extract were 7.6 ± 0.6 μg/mL, 3.9 ± 0.6 μg/mL, and 63.3 ± 5.4 μg/mL, while the stem extract had IC_50_ values of 17.3 ± 0.3 μg/mL, 5.8 ± 0.5 μg/mL, and 93.5 ± 5.5 μg/mL [18].

Regarding the antioxidant potential of various GR methanolic extracts, Ilić et al. examined two dry methanolic extracts from the aerial and underground parts of GR. The aerial parts extract demonstrated a superior antioxidant capacity in the DPPH test, with an SC_50_ value of 6.81 ± 0.16 μg/mL, compared to 7.54 ± 0.07 μg/mL for the underground parts. Furthermore, the findings revealed that the extract from the aerial parts was significantly richer in polyphenolic compounds, measuring 425.31 ± 3.37 mg GAE/g [53]. In the same regard, Ben Jemia and colleagues conducted a study on the antioxidant activity of a methanolic extract from the leaves of Tunisian GR. Using the DPPH method, they observed that this extract displayed strong antioxidant potential (IC_50_ = 19.98 ± 0.05 µg/mL), which was greater than that of vitamin C (IC_50_ = 12 ± 0.13 µg/mL). Regarding the reducing power of this extract, the obtained data showed an EC_50_ value of 20 ± 4.5 µg/mL, superior to vitamin C (40 µg/mL). The electron donors present in the methanolic extract significantly reduce free radicals [19]. Another 99% methanolic extract from GR leaves was investigated in terms of its antioxidant potential. The DPPH assay unveiled an IC_50_ value of 64.56 μg/mL, indicating that its free radical scavenging ability was significantly less effective than Trolox, the reference standard compound, which had an IC_50_ of 2.08 μg/mL [54].

Numerous studies have compared the radical scavenging potential of different organic or inorganic GR extracts. For example, Graça and his collaborators chemically characterized and bioevaluated various aqueous (infusion and decoction) and organic extracts (methanol, acetone, ethyl acetate, dichloromethane, and n-hexane) from Portuguese GR. The antioxidant potential of these extracts was assessed using the DPPH test, where the acetone, methanol, and aqueous extracts demonstrated comparable activity, with EC_50_ values ranging from 54 to 60 µg/mL. They also found that the acetone extract contained the highest amount of phenols (347 mg GAE/g extract) and flavonoids (53 mg CE/g extract), followed by the methanol and ethyl acetate extracts. The HPLC-DAD-ESI/MS analysis revealed that the acetone extract contained fourteen phenolic acid derivatives and six flavonoid glycosides, with geraniin being the most abundant compound (45 ± 1 mg/g extract) [10]. Another comprehensive study conducted by Neagu et al. aimed to determine the antioxidant capacity and phytochemical composition of various GR extracts obtained from leaves (6% and 10% aqueous, 50% and 70% ethanol). They observed that the 70% ethanolic extract demonstrated the highest efficacy against free radicals, achieving a DPPH inhibition rate of 90.37%. The TEAC values were 242.75 μmol Trolox/g for the DPPH method and 782.30 μmol Trolox/g for the ABTS method. An explanation for why the TEAC values obtained in the DPPH test are lower than those in the ABTS test is that the DPPH test is more specific, targeting only beta-substituted polyphenols that can neutralize the DPPH radical. A significant correlation was observed between high polyphenol content and increased antioxidant capacity. The extract with the highest levels of polyphenols was the 50% ethanol extract, which had a concentration of 4.21 mg/mL. In contrast, the 6% aqueous extract contained only 1.96 mg/mL of total polyphenols [16].

Although herbal medicine has been used since ancient times and its popularity continues to increase [55,56], medicinal plants may contain hazardous substances, including potentially toxic metals, which can pose health risks due to their toxicity, carcinogenicity, mutagenicity, and teratogenicity [56]. Furthermore, the bioaccumulation of heavy metals can have negative impacts on human health, leading to various toxic effects on different body tissues and organ systems [57].

On the other hand, an optimal level of essential heavy metals, such as cobalt (Co), copper (Cu), iron (Fe), manganese (Mn), molybdenum (Mo), nickel (Ni), and zinc (Zn), has a beneficial role in the growth, development, and improvement of the nutritional value of plants [58]. In addition, a proper balance of minerals is vital for maintaining normal human health and preventing disorders related to deficiencies and toxicities [59]. Therefore, screening for heavy metals in medicinal plants is important both to assess their safety for human consumption and to better understand their nutritional properties.

In this study, ten metals, including several with known toxic potential, were quantified in the dry extract of GR by atomic absorption spectroscopy. Four of these potentially hazardous heavy metals—arsenic (As), Co, lead (Pb), and cadmium (Cd)—were found to be under the detection limit.

Hasanović et al. [60] investigated GR populations from both heavy metal-contaminated and uncontaminated sites to assess their metal accumulation patterns and tolerance potential. The concentrations of heavy metals detected in plant samples were in the following order: Fe > Zn > Mn > Ni > Cu > chromium (Cr) > Cd > Co > Pb. Their results demonstrated that Fe concentrations in all plant samples ranged between 60 and 600 ppm, consistently exceeding the permissible threshold in vegetables. Furthermore, specimens originating from serpentine soils, which are characteristically rich in heavy metals, showed significantly elevated levels of Ni and Zn, also surpassing regulatory limits. Elevated heavy metal levels were associated with increased secondary metabolite production in GR, with phenolics significantly higher in plants from heavy metal-rich soils, suggesting an adaptive stress response.

Moreover, Fe was also found to be the most abundant element in the analyzed extract, with a concentration of 363.65 ± 4.18 μg/g, followed by Zn, Mn, Ni, and Cr. The concentration of Cu was below the detection limit.

Fe is a micronutrient that is found in abundance in the human body, with major functions in various biological processes, being an essential element for supporting life [61]. Fe deficiency, a major cause of anemia, is one of the most widespread nutritional deficiencies worldwide, affecting over 25% of the global population [59]. Fe absorption in the human body depends on its form; heme Fe from animal sources is more bioavailable than non-heme Fe found in plants, which unfortunately limits the effectiveness of plant-based Fe sources [62].

Zn is the second most abundant trace element in the body after Fe, being found in the highest proportion in bones and skeletal muscles. It is an essential micronutrient with a key role in several biological processes. However, the human body cannot store Zn in sufficient amounts; therefore, a regular dietary intake is vital to support its multiple functions [63,64]. In our study, the Zn concentration in the dry extract of GR was 24.25 μg/g, lower than the lowest value reported by Hasanović et al. [60], and may contribute to some extent to daily Zn intake.

Mn is an essential micronutrient for plant metabolism, intervening in photosynthesis, enzyme activation, and disease resistance. It is also an essential trace element for human nutrition, required in various enzymatic activities involved in metabolism, bone formation, and blood sugar regulation [65]. Both Mn deficiency and excess can cause negative health effects, overexposure to Mn being correlated with an increased risk of behavioral disorders in children and neurodegenerative diseases in adults [66]. The Adequate Intake (AI) levels for adults are 2.3 mg/day for men and 1.8 mg/day for women, with the tolerable upper intake level set at 11 mg/day [67]. Mn is considered toxic if it is ingested in doses higher than 40 mg/day [66]. Hasanović et al. [60] reported Mn concentrations in the range of 15.8–40.6 ppm in GR plant samples. In the present study, a lower content of Mn (10.8 ppm) was detected in the dry extract of GR, which may contribute to the daily requirement.

Ni is a naturally occurring transition metal, and for most plants it is considered an essential micronutrient required for improving plant development and growth, but at low concentrations (0.05–10 mg/kg dry weight) [68,69]. In humans, natural Ni deficiencies practically do not occur, since the usual daily dietary intake of 25 to 300 μg/day is more than three times higher than the daily requirement [70]. In our dry extract of GR, the Ni content was 0.85 μg/g, which is significantly lower than the levels between 1.89 and 38.95 ppm reported by Hasanović et al. [60]. However, Ni contamination has become a major environmental problem, especially in developing countries where anthropogenic activities contribute significantly to its accumulation in soil and water, consequently affecting the food production systems [71].

Cr effects depend on its oxidation state. Cr(III) is an essential nutrient playing a major role in glucose and fat metabolism, while Cr(VI) is known to be carcinogenic and comes mostly from industrial and anthropogenic activities [72]. Cr(III) is found in many foods and dietary supplements, but is poorly assimilated by the body [72]. The AI levels of Cr were set at 35 μg/day for men and 25 μg/day for women, respectively [67]. The total Cr content determined in our GR dry extract was 0.75 μg/g. Similar values, but some even higher, were reported by Hasanović et al. [60].

Within this study, the antimicrobial activity of GR extract was evaluated using the disk diffusion method and MIC determination against selected bacterial strains. However, its effectiveness against *Pseudomonas aeruginosa* was notably weaker. The MIC values of 25 µg/mL suggest a consistent inhibitory concentration across most tested bacteria.

Several studies have evaluated the antibacterial effect of different GR extracts.

Using the plate-hole diffusion assay, Alhage et al. investigated the antibacterial activity of different extracts (dichloromethane, methanol, and aqueous crude extracts) of Lebanese GR. They found that the dichloromethane extract from the leaves was the most effective against *S. aureus*, achieving an inhibition diameter of 8 mm at a concentration of 1 mg/mL. In comparison, the leaf methanolic extract also resulted in an 8 mm inhibition diameter but required a concentration that was ten times higher. Regarding the Gram-negative tested strains (*P. aeruginosa* and *E. coli*), GR extracts displayed weak effectiveness [15]. Another study, conducted by Świątek et al., evaluated the antimicrobial activity of 4 different extracts obtained from aerial parts of Polish GR (methanolic, ethanolic, aqueous, and hexane). It was proven that all 4 extracts presented activity against all Gram-positive bacterial strains, the hexane extract being superior to the other extracts according to MIC values (0.06–0.5 mg/mL). The ethanolic extract was found to be effective against methicillin-resistant *S. aureus*, with a MIC value of 2 mg/mL [6].

Ilić and his collaborators obtained a methanolic extract (in a plant: solvent ratio of 1:10) from the aerial parts of GR from Southeastern Serbia. They subsequently tested the extracts by the broth microdilution method against several standard strains and clinical isolates of Gram-positive and Gram-negative bacteria. The data obtained indicated MIC values from 25 to >200 μg/mL, the most sensitive species being *E. faecalis* ATCC 29212 and *E. coli* ATCC 10536. Against *S. aureus* ATCC 6538, the extract from the aerial parts displayed a MIC value of 100 μg/mL [53]. However, the ethanolic extract tested within this study was found to be more potent than the two previously mentioned extracts (MIC = 25 μg/mL).

The antibacterial activity of *Geranium* species can be attributed to hydrolyzable tannins, which are also part of the polyphenol class. It is assumed that these compounds can alter the structure of the cell wall and inhibit glucosyltransferase. Furthermore, polyphenolic compounds, due to their antioxidant effect, can prevent oxidative stress encountered in bacterial infections [53].

To evaluate the antiproliferative potential of the ethanolic extract of GR from Romania against the malignant A253 cell line, the standard MTT method was employed (72 h of incubation). The findings indicated a dose-dependent antiproliferative effect, with the most significant cytotoxicity (19.2%) being observed at a concentration of 200 μg/mL. The therapeutic anticancer potential of GR, although recognized in traditional medicine, has not been extensively investigated mechanistically. Various aqueous or organic extracts were effective against several malignant cell lines (HeLa, HepG2, MCF-7, NCIH460) [52].

Paun et al. investigated the cytotoxic effects of aqueous and 50% ethanolic extracts of Romanian GR leaves, concentrated using ultra- and microfiltration, against a human laryngeal cancer cell line (Hep-2p), with normal monkey kidney cells used as comparison. They found that these extracts exerted a good cytotoxic effect against cancer cells and minor toxicity against healthy ones. The 50% ethanolic microfiltrate extract showed a cytotoxicity percentage of 22.2% against malignant cells (similar to the cytotoxicity observed in the present study) and 8.1% against normal cells. In contrast, the aqueous microfiltrate extract exhibited a lower cytotoxic effect, with values of 7.3% for cancer cells and 3.9% for normal cells [42]. Likewise, Neagu et al. examined the antiproliferative activity of an aqueous GR extract obtained through microfiltration against the HEp-2 human laryngeal cancer cell line, using 4 concentrations (100, 500, 1000, and 2000 mg/mL) and 2 periods of incubation (24 and 48 h). The viability of the cells decreased in a manner that depended on both the dose and the duration of exposure. At the highest tested concentration (2000 mg/mL), after 48 h of incubation, the extract resulted in a cell viability of 2.8%, which indicates that the GR extract has a significant antiproliferative capacity [20]. Catarino et al. conducted a study to evaluate the toxicity of two aqueous extracts obtained by decoction from the leaves and stems of GR, sourced from Portugal. They tested these extracts against two healthy cell lines: HepG-2 (ATCC HB-8065) hepatocytes and RAW 264 macrophages. The MTT assay revealed that the viability of hepatocytes was unchanged by these two extracts at the tested concentrations (25, 50, 75, and 100 μg/mL), while the leaf extract was associated with significant cytotoxic effects on lipopolysaccharide-stimulated macrophages only at the highest tested concentration [18].

A comprehensive study conducted by Graca et al. evaluated the cytotoxic effects of various Portuguese GR extracts (aqueous, acetone, ethyl acetate, methanol, and n-hexane) derived from the whole plant against several cancer cell lines (MCF-7, HeLa, HepG2, and NCI-H460) and one non-cancer cell line (PLP2). All extracts included in this study demonstrated cytotoxicity against the malignant cell lines. Of all, the acetone extract proved to be the most potent one, presenting GI_50_ values ranging between 57 and 60 μg/mL on malignant cells, while also having a toxic effect on healthy liver cells (GI_50_ = 176 μg/mL). The two aqueous extracts (infusion and decoction) exhibited GI_50_ values similar to those of the acetone extract against the breast cancer cell line (MCF-7) and hepatocellular carcinoma (HepG2), without displaying hepatotoxicity at the administered dose of 400 μg/mL [10]. In this context, the high polyphenolic content was directly linked to increased anticancer activity, as this group of phytocompounds is known for its ability to inhibit the growth of cancer cells [3,73]. Another study designed by Świątek et al. aimed to evaluate the cytotoxic activity of four organic extracts from the aerial parts of the Polish GR plant (aqueous, methanol, ethyl acetate, and hexane) using the MTT assay. These extracts were tested against both normal (VERO) and cancer cell lines, specifically pharyngeal cancer cell lines (Detroit 562 and FaDu) and colon carcinoma cell line (RKO). After 72 h of incubation, the methanolic extract demonstrated the highest toxicity against VERO cells among all extracts (CC_50_ = 187.17 μg/mL), while the aqueous extract was found to be the least toxic against all the tested cell lines (CC_50_ = 145.83–381.1 μg/mL). GR ethyl acetate and hexane extracts possessed significant and selective anticancer potential, especially against FaDu and RKO cell lines (CC_50_ ethyl acetate = 63.36–63.63 μg/mL; CC_50_ hexane = 98.91–109.23 μg/mL) [6].

Regarding other *Geranium* species, Pirvu et al. investigated a Romanian *Geranium pratense* concentrated ethanolic extract from aerial parts (containing 5 mg GAE/mL). Within the MTS assay, it was observed that GR ethanolic extract determined rather a stimulation of cell growth than an inhibition on normal cells derived from the mammary gland (MCF-12A) (cell viability increased by up to 15% during the cytotoxic test), but also for the human cancer breast cells (BT-20) (7% and 8% increases in cell viability at 24 h and 48 h) [74]. In the same vein, Romero-Benavides and colleagues studied the cytotoxic potential of an Ecuadorian *Geranium diffusum* methanolic extract from aerial parts and its solvent fractions against three colon carcinoma cell lines (HCT-116, SW612-B3, and RKO). The MTS test (48 h of incubation, sample concentration of 50 μg/mL) revealed that the methanolic extract and its fractions, except the ethyl acetate fraction, did not significantly reduce the viability of SW613-B3 cells, maintaining the viability over 70%. In contrast, the ethyl acetate fraction exhibited a strong cytotoxic effect on the same cell line, with an IC_50_ value of 44.47 ± 1.02 μg/mL. In the case of the HCT-116 and RKO cell lines, none of the tested samples caused a decrease in cell viability below the 50% threshold [75].

Concerning the effects of GR extracts on cell and nuclear morphology, as well as the colony formation process, the research team conducted by Neagu aimed to investigate the effect of GR aqueous extracts obtained by micro- and ultrafiltration on HEp-2 human laryngeal carcinoma cellular morphology. After 48 h of treatment with 500 and 1000 μM/mL of these GR extracts, the morphology of cancerous cells was significantly altered. Microscopic images revealed condensed nuclei, granular cytoplasm, and the absence of dividing cells. Furthermore, only 40% of the well area was occupied by the treated cells as opposed to over 95% by the untreated cells [20].

One of the main factors contributing to cancer development is the increased presence of reactive oxygen species (ROS). Oxidative stress plays an important role in regulating cell survival and apoptosis [76]. After treatment with GR extract, a dose-dependent increase in ROS production was observed. The results are consistent with previously reported oxidative stress-mediated cytotoxic effects of plant-derived compounds [77]. The use of phytocompounds with antioxidant properties may help reduce oxidative stress. In this regard, Huang et al. investigated the ability of geraniin, a prominent tannin in *Geranium* species, to attenuate hydrogen peroxide-induced oxidative stress in cells in vitro. Hoechst 33342 staining showed that geraniin-pretreated bone marrow-derived mesenchymal stem cells (1, 5, 10, 20 μM) exhibited significantly less nuclear damage than untreated cells. In contrast, untreated cells displayed marked alterations in nuclear morphology, including reduced nuclear size and fragmentation. These results indicate that geraniin may help reduce apoptosis induced by H_2_O_2_ [78]. Additionally, Chen et al. studied the impact of geraniin on NF-κB expression. The immunofluorescence assay showed that supplementing C666-2 nasopharyngeal cancer cells with 5 and 10 μM/mL geraniin significantly reduced NF-κB expression compared to the control or C666-2 cells. As it is well known that proteins transcribed by NF-κB may stimulate tumor development by inhibiting the apoptotic process, the decrease in their expression represents a beneficial effect of this tannin [79]. Lee et al. also found that geraniin has therapeutic potential against A2058 human melanoma cells, as it can induce apoptosis in a dose- and time-dependent manner. Furthermore, it was stated that geraniin possesses pro-apoptotic potential by mediating caspase-3 signaling pathways. These findings suggest that geraniin could have both curative and prophylactic roles [50,80].

Within another study, Lee et al. aimed to elucidate the influence of *Geranium thunbergii* 80% methanolic extract against six cell lines of gastric cancer (SNU-216, SNU-601, SNU-668, MKN-28, YCC-2, and AGS). The extract (50 to 500 μM/mL) inhibited cell proliferation in a dose-dependent manner, as demonstrated by the WST-1 assay. This effect was observed in all tested cell lines. Additionally, Western blot analysis indicates that the apoptotic death of the SNU-668 and YCC-2 cells is associated with the activation of caspase 3 [81].

The anticancer potential of GR extracts may also be attributed to their content of polyphenolic compounds. In particular, gallic and ellagic acids have been reported to contribute to the suppression of carcinogenic processes. Additionally, flavonoids such as kaempferol and quercetin demonstrate anticancer properties by being cytotoxic against various types of cancer cells. They achieve this through the induction of apoptosis, increasing oxidative stress, and interactions with key signaling pathways associated with cancer (MAPK, NF-κB, and PI3K/Akt) [6,79,82]. Unlike most previous studies on *G. robertianum*, which focused primarily on total phenolics or general antioxidant activity, our work integrates chemical, safety-related, and cellular data obtained from the same extract. The correlation between the phytochemical profile, ROS induction, and caspase activation in A253 cells provides new mechanistic evidence supporting the pro-apoptotic potential of this plant.

## 5. Conclusions

The findings of the present study contribute to a wider characterization and evaluation of GR ethanolic extract obtained from the aerial parts of the plant. The phytochemical characterization indicated that the GR extract had high levels of phenolic compounds and flavonoids. The antioxidant activity studied by means of three different colorimetric assays showed a high value, the highest antioxidant capacity being obtained at the ABTS assay. The inorganic elements evaluation indicated that Fe was the most abundant element in the analyzed extract, followed by Zn, Mn, Ni, and Cr, whereas potentially hazardous heavy metals, As, Co, Pb, and Cd, were found to be under the detection limit. The GR extract demonstrated a moderate inhibitory effect on both Gram-positive and Gram-negative bacterial strains. On A253 human salivary gland carcinoma cells, GR ethanolic extract had a dose-dependent antiproliferative effect, with the most significant cytotoxicity being observed at the highest concentration tested.

## Figures and Tables

**Table 1:** Program parameters (T—temperature, t—time, P—power) for microwave acidic digestion.

Digestion parameters	**T1**	**t1**	**p1**	**T2**	**t2**	**p2**	**T3**	**t3**	**p3**
	160 °C	15 min	80%	210 °C	15 min	90%	Gradual decrease in temperature	15 min	0

**Table 2:** Operating conditions for the metal analysis by atomic absorption spectroscopy.

No	Metal	Wavelength, λ (nm)	Calibration Range (μg/L)	Calibration Curve Abs = f(conc.)	R^2^
1	Fe	248.3	0–14.4	y = 0.021707 + 0.010187x	0.9988
2	Cu	324.8	0–18.0	y = 0.038506 + 0.048577x	0.9931
3	Ni	232.0	0–31.5	y = 0.115634 + 0.006821x	0.9998
4	Mn	279.5	0–3.36	y = 0.007792 + 0.112496x	0.9925
5	As	193.7	0–52.8	y = −0.001185 + 0.001544x	0.9927
6	Al	309.3	0–52.8	y = 0.006978 + 0.001749x	0.9971
7	Zn	213.9	0–8.0	y = 0.071658 + 0.092202x	0.9827
8	Co	240.7	0–21.6	y = 0.007448 + 0.008841x	0.9974
9	Pb	283.3	0–38.0	y = 0.004606 + 0.004331x	0.9959
10	Cr	357.9	0–20.0	y = 0.013314 + 0.018746x	0.9932
11	Cd	228.8	0–2.0	y = 0.007384 + 0.100405x	0.9903

**Table 3:** TPC and TFC of GR ethanolic extract.

	TPC (mg GAE/g Dried Extract)	TFC (mg QE/g Dried Extract)
GR	191.7 ± 5.8	117.4 ± 8.3

**Table 4:** Phytochemical composition of GR ethanolic extract.

Chemical Class	Compound	Concentration (Mean ± SD, *n* = 3)
Hydroxycinnamic acids (mg/g dried extract)	Caffeic acid	<LOQ *
	Chlorogenic acid	0.77 ± 0.023
	4-O-Caffeoylquinic acid	<LOQ *
	*p*-Coumaric acid	0.627 ± 0.031
	Ferulic acid	0.681 ± 0.027
Hydroxybenzoic acids (mg/g dried extract)	Syringic acid	0.0039 ± 0.0001
	Gallic acid	43.442 ± 3.04
	Protocatechuic acid	1.756 ± 0.122
	Vanillic acid	0.0252 ± 0.002
	Gentisic acid	<LOQ *
Flavanols (mg/g dried extract)	Procyanidin B1	0.0069 ± 0.0005
Flavonols (mg/g dried extract)	Hyperoside	4.272 ± 0.299
	Isoquercitrin	2.694 ± 0.107
	Rutin	<LOQ *
	Quercitrin	0.291 ± 0.02
	Quercetin	1.224 ± 0.036
	Kaempferol	<LOQ *
Flavones (mg/g dried extract)	Luteolin	<LOQ *
	Apigenin	0.103 ± 0.003
Tocopherols (mg/g dried extract)	δ-Tocopherol	0.00170 ± 0.00001
Sterols (mg/g dried extract)	Stigmasterol	0.223 ± 0.008
	β-Sitosterol	5.391 ± 0.269
	Campesterol	0.261 ± 0.023

* <LOQ—below the limit of quantification for the analytical method.

**Table 5:** Antioxidant activity of GR ethanolic extract by three methods.

	Antioxidant Activity (mmol TE/g Dried Extract)		
	DPPH	ABTS	FRAP
GR	2.091 ± 0.019	3.159 ± 0.121	2.061 ± 0.002

**Table 6:** The concentration of detected metals in the GR ethanolic extract.

Sample	Element Concentration (μg/g) *									
	Fe	Cu	Ni	Mn	As	Zn	Co	Pb	Cr	Cd
GR	363.65 ± 4.18	ND **	0.85 ± 0.03	10.80 ± 0.17	ND	24.25 ± 0.30	ND	ND	0.75 ± 0.04	ND

* mean of six determinations ± standard deviation. ** ND: not detected (below limit of detection).

**Table 7:** Antimicrobial activity of GR ethanolic extract.

Bacterial Strains	Sample	Disk Diffusion Method (Inhibition Zones in mm)	MIC (µg/mL)
*Streptococcus mutans* ATCC 35668	GR	17	25
	Levofloxacin	19	NA *
*Streptococcus pyogenes* ATCC 19615	GR	18	25
	Levofloxacin	20	NA *
*Staphylococcus aureus* ATCC 25923	GR	17	25
	Levofloxacin	20	NA *
*Escherichia coli* ATCC 25922	GR	17	25
	Levofloxacin	21	NA *
*Pseudomonas aeruginosa* ATCC 27853	GR	7	-
	Levofloxacin	20	NA *

* NA = not applicable.

## Data Availability

The data supporting the findings of the study are available within the article.

## Supplementary Materials

The following supporting information can be downloaded at https://www.mdpi.com/article/10.3390/antiox15030296/s1, Supplementary Material includes the UV chromatograms for individual polyphenols (method 1) and the total ion chromatograms (TIC) for the additional phenolic acids and catechins (method 2), tocopherols, and sterols. Additionally, the supplementary tables provide a detailed report of the retention times, molecular formulas, molecular ions, and fragmentation patterns for all monitored compounds.

## Abbreviations

The following abbreviations are used in this manuscript:

A253	Human submaxillary salivary gland carcinoma cell line
ABTS	2,2′-azino-bis(3-ethylbenzthiazoline-6-sulphonic acid)
AI	Adequate Intake
APCI	Atmospheric pressure chemical ionization
As	Arsenic
ATCC	American Type Culture Collection
BHT	Butylated hydroxytoluene
Cd	Cadmium
CLSI	Clinical Laboratory and Standard Institute
Co	Cobalt
Cr	Chromium
Cu	Copper
DPPH	1,1-diphenyl-dipicrylhydrazyl
F-actin	Filamentous actin
FBS	Fetal bovine serum
Fe	Iron
FRAP	Ferric Reducing Antioxidant Power assay
GAE	Gallic acid equivalents
GR	*Geranium robertianum* L.
HEp-2	Human epidermoid laryngeal carcinoma cell line
MH	Mueller-Hinton
MIC	Minimum inhibitory concentration
Mn	Manganese
Mo	Molybdenum
MRM	Multiple-reaction monitoring
Ni	Nickel
Pb	Lead
PBS	Phosphate-buffered saline
QE	Quercetin equivalents
ROS	Reactive oxygen species
SLS	Sodium lauryl sulphate
TE	Trolox equivalent
TEAC	Total equivalent antioxidant capacity
TPC	Total phenolic content
TPTZ	2,4,6-tris(2′-pyridyl)-1,3,5-triazine
UAE	Ultrasound-assisted extraction
Zn	Zinc
