# Supplementary material for: Phytochemical Characterization and Biological Assessment of Geranium robertianum L. Ethanolic Extract on Human Salivary Gland Carcinoma Cells
Source: Antioxidants (Basel). 2026 Feb 27;15(3):296. doi: 10.3390/antiox15030296 (PMC13023475; doi:10.3390/antiox15030296)
Supplement: Supplementary file 1 [file antioxidants-15-00296-s001.zip › antioxidants-4137435-supplementary.pdf]

Sterols						
No.	Compound name	Molecular formula	Retention time (min)	Analysis mode (ion source, polarity, transition type)	Molecular ion $[M-H_2O+H]^+$	Daughter ions
1	Ergosterol	$C_{28}H_{44}O$	2.4	APCI+, MRM	379	253, 309
2	Stigmasterol	$C_{29}H_{48}O$	4.0	APCI+, MRM	395	297, 311
3	Campesterol	$C_{28}H_{48}O$	4.6	APCI+, MRM	383	257, 287
4	β-Sitosterol	$C_{29}H_{50}O$	5.4	APCI+, MRM	397	243, 257

Tocopherols						
No.	Compound name	Molecular formula	Retention time (min)	Analysis mode (ion source, polarity, transition type)	Molecular ion $[M-H]^-$	Daughter ions
1	δ-Tocopherol	$C_{27}H_{46}O_2$	3.2	APCI-, MRM	401	135
2	γ-Tocopherol	$C_{28}H_{48}O_2$	4.05	APCI-, MRM	415	149
3	α-Tocopherol	$C_{29}H_{50}O_2$	5.1	APCI-, MRM	429	163

Hydroxybenzoic Acids (compounds based on a C_6-C_1 structure)						
No.	Compound name	Molecular formula	Retention time (min)	Analysis mode (ion source, polariry, transition type)	Molecular ion $[M-H]^-$	Daughter ions
1	Gallic acid	$C_7H_6O_5$	1.5	ESI-, MRM	169	125
2	Protocatechuic acid	$C_7H_6O_4$	2.9	ESI-, MRM	153	109
3	Vanillic acid	$C_8H_8O_4$	6.3	ESI-, MRM	167	152
4	Syringic acid	$C_9H_{10}O_5$	8.4	ESI-, MRM	197	182
5	Gentisic acid	$C_7H_6O_4$	2.72	ESI-, MRM	153	109

**Hydroxycinnamic Acids**<br>(compounds based on a C_6-C_3 structure and their various derivatives)						
No.	Compound name	Molecular formula	Retention time (min)	Analysis mode<br>(ion source, polariry, transition type)	Molecular ion<br>$[M-H]^-$	Daughter ions
1	Caftaric acid	$C_{13}H_{12}O_9$	2.15	ESI-, MRM	311	179
2	Caffeic acid	$C_9H_8O_4$	5.85	ESI-, MRM	179	135
3	Chlorogenic acid	$C_{16}H_{18}O_9$	6.63	ESI-, MRM	353	191
4	4-O-Caffeoylquinic acid	$C_{16}H_{18}O_9$	7.0	ESI-, MRM	353	173
5	*p*-Coumaric acid	$C_9H_8O_3$	9.15	ESI-, MRM	163	119
6	Ferulic acid	$C_{10}H_{10}O_4$	12.43	ESI-, MRM	193	134
7	Sinapic acid	$C_{11}H_{12}O_5$	14.67	ESI-, MRM	223	149

**Flavanols**<br>(monomeric flavan-3-ols and their polymers)						
No.	Compound name	Molecular formula	Retention time (min)	Analysis mode<br>(ion source, polariry, transition type)	Molecular ion<br>$[M-H]^-$	Daughter ions
1	Epigallocatechin	$C_{15}H_{14}O_7$	5.8	ESI-, SIM	305	305
2	Catechin	$C_{15}H_{14}O_6$	6	ESI-, SIM	289	289
3	Epigallocatechin gallate	$C_{22}H_{18}O_{11}$	8.2	ESI-, SIM	457	457
4	Epicatechin	$C_{15}H_{14}O_6$	8.7	ESI-, SIM	289	289
5	Procyanidin B1	$C_{30}H_{26}O_{12}$	2.5	ESI-, MRM	577	407; 425; 451
6	Procyanidin B2	$C_{30}H_{26}O_{12}$	5.1	ESI-, MRM	577	407; 425; 451
7	Procyanidin C1	$C_{45}H_{38}O_{18}$	7.1	ESI-, MRM	865	407; 425; 451
8	Procyanidin A1	$C_{30}H_{24}O_{12}$	7.7	ESI-, MRM	575	407; 423; 447

**Flavonols**<br>(aglycones and their various glycosides)						
No.	Compound name	Molecular formula	Retention time (min)	Analysis mode<br>(ion source, polariry, transition type)	Molecular ion<br>$[M-H]^-$	Daughter ions
1	Hyperoside	$C_{21}H_{20}O_{12}$	18.96	ESI-, MRM	463	301
2	Isoquercitrin	$C_{21}H_{20}O_{12}$	19.9	ESI-, MRM	463	301
3	Rutin	$C_{27}H_{30}O_{16}$	20.4	ESI-, MRM	609	301
4	Myricetin	$C_{15}H_{10}O_{8}$	21.1	ESI-, SIM	317	317
5	Fisetin	$C_{15}H_{10}O_{6}$	22.8	ESI-, SIM	285	285
6	Quercitrin	$C_{21}H_{20}O_{11}$	23.26	ESI-, MRM	447	301
7	Kaempferitrin	$C_{27}H_{30}O_{14}$	25.6	ESI-, MRM	577	285
8	Quercetin	$C_{15}H_{10}O_{7}$	26.82	ESI-, SIM	301	301
9	Kaempferol 3-rhamnoside	$C_{21}H_{20}O_{10}$	27.4	ESI-, MRM	431	285
10	Patuletin	$C_{16}H_{12}O_{8}$	28.74	ESI-, SIM	331	331
11	Kaempferol	$C_{15}H_{10}O_{6}$	31.73	ESI-, SIM	285	285

**Flavones**<br>(compounds with a double bond between C_2 and C_3 and a ketone group at C_4)						
No.	Compound name	Molecular formula	Retention time (min)	Analysis mode<br>(ion source, polariry, transition type)	Molecular ion<br>$[M-H]^-$	Daugther ions
1	Vitexin	$C_{21}H_{20}O_{10}$	18	ESI-, MRM	431	311
2	Vitexin 2-O-rhamnoside	$C_{27}H_{30}O_{14}$	19.4	ESI-, MRM	577	431
3	Luteolin	$C_{15}H_{10}O_{6}$	29.24	ESI-, SIM	285	285
4	Apigenin	$C_{15}H_{10}O_{5}$	33.24	ESI-, SIM	269	269
